# Updated description of *Atheniella* (Mycenaceae, Agaricales), including three new species with brightly coloured pilei from Yunnan Province, southwest China

**DOI:** 10.3897/mycokeys.81.67773

**Published:** 2021-07-14

**Authors:** Yupeng Ge, Zewei Liu, Hui Zeng, Xianhao Cheng, Qin Na

**Affiliations:** 1 Shandong Key Laboratory of Edible Mushroom Technology, School of Agriculture, Ludong University, Yantai 264025, China Ludong University Yantai China; 2 Institute of Edible Fungi, Fujian Academy of Agricultural Sciences; National and Local Joint Engineering Research Center for Breeding & Cultivation of Features Edible Fungi, Fuzhou 350014, China Institute of Edible Fungi, Fujian Academy of Agricultural Sciences Fuzhou China

**Keywords:** new taxon, polygenes, taxonomy, white basidiospores

## Abstract

An updated description of the genus *Atheniella*, combining macro- and micromorphological characters that elaborate on the original generic characterisation, is presented. *Atheniella* is characterised by a brightly coloured pileus, all tissues inamyloid and pileipellis covered with simple to branched excrescences. Previously, nine *Atheniella* species were known globally, of which three species were accepted in China. Three newly-recognised species classified in the genus are here formally described from Yunnan Province: *Atheniella flavida***sp. nov.**, *A. rutila***sp. nov.** and *A. taoyao***sp. nov.** The new species are characterised by a yellow, orange, pink or red pileus, fusiform cheilocystidia and pleurocystidia, non-smooth pileipellis, stipitipellis smooth or with cylindrical ornamentation, caulocystidia fusiform or subglobose, if present and all tissues inamyloid. Morphological descriptions, photographs, line drawings and comparisons with closely-related taxa are presented for the new species. A phylogenetic analysis of sequence data for the rDNA internal transcribed spacer region and nuclear large ribosomal subunit (ITS + nLSU) supported that *Atheniella* is resolved as monophyletic and also supported the taxonomic recognition of the new species. A key to the 12 species of *Atheniella* is also provided.

## Introduction

The genus *Atheniella* Redhead, Moncalvo, Vilgalys, Desjardin & B.A. Perry is a small mycenoid genus, formerly treated as *Mycena* (Pers.) Roussel sect. Adonideae (Fr.) Quél., that was elevated to genus rank by Redhead et al. (2012). *Atheniella* is characterised macroscopically by its habit resembling that of *Mycena* owing to the small basidiomata, white lamellae, hollow stipe and is saprophytic on rotten wood or plant debris (Kühner 1938; Smith 1947; Redhead et al. 2012; Aronsen and Læssøe 2016). Redhead et al. (2012) noted that the brightly coloured pileus (e.g. yellow, orange, pink or red) and all tissues unreactive in Melzer’s Reagent are diagnostic characters that distinguish *Atheniella* from *Mycena*. Given the change in taxonomic rank, formalisation of new combinations in *Atheniella* was required for species formerly classified in Mycena sect. Adonideae (Grgurinovic 2003; Redhead et al. 2012; Aravindakshan and Manimohan 2015; Gminder and Böhning 2016; Lehmann and Lüderitz 2018, 2019). In previous publications, nine taxa were recognised in *Atheniella*, comprising three new species and six new combinations, of which *Atheniella adonis* (Bull.) Redhead, Moncalvo, Vilgalys, Desjardin & B.A. Perry is the type species (Grgurinovic 2003; Redhead et al. 2012; Aravindakshan and Manimohan 2015; Gminder and Böhning 2016; Lehmann and Lüderitz 2018, 2019). An infrageneric classification for *Atheniella* has not been proposed since the genus was established (Redhead et al. 2012).

Previous taxonomic studies of *Atheniella* are incomplete because of insufficient species representation and a lack of phylogenetic evidence and only four taxa of *Atheniella* have been included in phylogenetic studies (Moncalvo et al. 2002; Matheney et al. 2006). Based on a phylogenetic reconstruction for more than 800 euagaric taxa derived from a nuclear ribosomal large subunit RNA gene (nLSU) sequence dataset, Moncalvo et al. (2002) established the Mycenaceae (Clade 47) to include 10 genera, including *Mycena*. However, *Mycena aurantiidisca* (Murrill) Murrill (≡ *Atheniella aurantiidisca* (Murrill) Redhead, Moncalvo, Vilgalys, Desjardin & B.A.Perry) and *Mycena adonis* (Bull.) Gray (≡ *Atheniella adonis*) were separated from the Mycenaceae to form an independent lineage termed the “adonis” clade (Clade 26). Matheney et al. (2006) agreed with Moncalvo et al. (2002) in the establishment of the Mycenaceae and that the “adonis” group should be excluded from *Mycena*, but differed in the phylogenetic placement of *Atheniella* spp. *Mycena amabillissima* (Peck) Sacc. (≡ *Atheniella amabillissima* (Peck) Redhead, Moncalvo, Vilgalys, Desjardin & B.A. Perry) and *M. aurantiidisca* (≡ *Atheniella aurantiidisca*) was placed in the “hydropoid” subclade of the Marasmioid clade (IV) (Matheney et al. 2006).

Species of *Atheniella* are widespread in temperate regions, but also distributed in the tropical zone (Smith 1935a, b, c, 1937, 1939; Maas Geesteranus 1980, 1990, 1992a, b; Redhead 1984; Perry 2002; Grgurinovic 2003; Robich 2003; Uehling et al. 2012; Aravindakshan and Manimohan 2015; Osono 2015; Aronsen and Læssøe 2016). Previous studies of *Atheniella* Kühner ex Singer during the last century focused on species distributed in Europe and North America (Murrill 1916; Tyler 1991; Emmett 1992; Gyosheva and Ganeva 2004; Friedrich 2006; Miersch and Karasch 2011; Miersch 2013; Pérez-De-Gregorio 2015; Norvell 2016; Aime et al. 2018). In contrast, few investigations of *Atheniella* taxa in Australia and Asia have been conducted. However, progress in clarifying the relationship between *Mycena* and *Atheniella* has been achieved in recent years (Miyamoto et al. 1998, 2000; Grgurinovic 2003; Aravindakshan and Manimohan 2015; Na 2019; Na and Bau 2018, 2019a, b).

Three *Atheniella* species, namely *A. adonis*, *A. aurantiidisca* and *A. flavoalba* (Fr.) Redhead, Moncalvo, Vilgalys, Desjardin & B.A. Perry, were previously recognised in China (Bau and Liu 2011; Li et al. 2015; Na 2019). During our ongoing research on *Mycena* s.l., three new mycenoid species belonging to *Atheniella* were discovered in Yunnan Province, southwest China and are formally described here as *A. flavida* Q. Na & Y.P. Ge, *A. rutila* Q. Na & Y.P. Ge and *A. taoyao* Q. Na & Y.P. Ge. In addition, the generic morphological description of *Atheniella* is updated and a key for identification of the 12 species of *Atheniella* currently known is provided.

## Materials and methods

### Morphological examination

Macroscopic descriptions were prepared, based on freshly-collected specimens, whereas micromorphological descriptions relied on dried material. In the descriptions, colour abbreviations follow Kornerup and Wanscher (1978). Microscopic observations were conducted on dried specimens mounted in 5% potassium hydroxide (KOH) and stained with Congo red when necessary. Melzer’s Reagent was used to test whether spores and tissues were amyloid (Horak 2005). Twenty mature basidiospores from each basidiocarp were measured in lateral view and one or two basidiocarps were examined per specimen. The basidiospore dimensions were recorded; the notation [*a*/*b*/*c*] used at the beginning of each basidiospore description indicates that *a* basidiospores from *b* basidiocarps of *c* specimens were measured. Measured dimensions (length × width) are presented as (d) e–**f**–g (h) × (i) j–**k**–l (m), where *d* is the minimum length, *e*–*g* represents the range of at least 90% of values, **f** is the average length and *h* is the maximum length; width (*i*–*m*) is expressed in the same manner. In addition, Q is the length: width ratio of a spore and **Q** ± SD is the average **Q** of all basidiospores ± the sample standard deviation. Authority abbreviations follow those used in Index Fungorum (https://www.indexfungorum.org). Voucher specimens have been deposited in the Fungarium of the Fujian Academy of Agricultural Sciences (**FFAAS**), China.

### DNA extraction, PCR amplification and DNA sequencing

Genomic DNA was extracted from tiny pieces of lamellae using the NuClean Plant Genomic DNA Kit (Kangwei Century Biotechnology Co., Beijing, China). The internal transcribed spacer (ITS) region and the nuclear large subunit (nLSU) of rDNA were amplified with the primer pairs ITS1/ITS4 and LROR/LR7, respectively (White et al. 1990; Hopple and Vilgalys 1999). The PCR thermocycling protocol (for both ITS and nLSU) was 94 °C for 4 min, followed by 34 cycles of 94 °C for 45 sec, 52 °C for 45 sec and 72 °C for 1 min and final extension for 10 min at 72 °C. The new sequences were submitted to GenBank (Table 1). The nBLAST tools (http://blast.ncbi.nlm.nih.gov/Blast.cgi) were used to compare the sequence identity with sequences in the NCBI databases. The GenBank accession numbers for the ITS and nLSU sequences are as follows: *Atheniella flavida* (MW969653–MW969654; MW969665), *A. rutila* (MW969658–MW969659; MW969668) and *A. taoyao* (MW969655–MW969657; MW969666–MW969667).

**Table 1.** Sequenced specimens used in phylogenetic analysis. New species are marked in bold.

No.	Taxa	Voucher	Locality	ITS Sequences ID	nLSU Sequences ID	Reference
1	*Atheniella adonis* (Bull.) Redhead, Moncalvo, Vilgalys, Desjardin & B.A. Perry	H6036863	FINLAND	MW540691	–	Unpublished
2	*A. adonis* (Bull.) Redhead, Moncalvo, Vilgalys, Desjardin & B.A. Perry	1058	CANADA	KJ705189	–	Unpublished
3	*A. adonis* (Bull.) Redhead, Moncalvo, Vilgalys, Desjardin & B.A. Perry	DAOM174885	–	–	AF261361	Moncalvo et al. (2002)
4	*A. amabillissima* (Peck) Redhead, Moncalvo, Vilgalys, Desjardin & B.A. Perry	AFTOL–ID 1686	USA	DQ490644	DQ457691	Matheney et al. (2006)
5	*A. amabillissima* (Peck) Redhead, Moncalvo, Vilgalys, Desjardin & B.A. Perry	TUR183733	FINLAND	MW540719	–	Unpublished
6	*A. amabillissima* (Peck) Redhead, Moncalvo, Vilgalys, Desjardin & B.A. Perry	BD–2020a	FINLAND	MW540733	–	Unpublished
7	*A. aurantiidisca* (Murrill) Redhead, Moncalvo, Vilgalys, Desjardin & B.A. Perry	UBC: F15202	CANADA	DQ384585	–	Unpublished
8	*A. aurantiidisca* (Murrill) Redhead, Moncalvo, Vilgalys, Desjardin & B.A. Perry	AFTOL–ID 1685	USA	DQ490646	DQ470811	Matheney et al. (2006)
9	*A. aurantiidisca* (Murrill) Redhead, Moncalvo, Vilgalys, Desjardin & B.A. Perry	UBC: F33062	CANADA	MF908459	–	Unpublished
10	*A. aurantiidisca* (Murrill) Redhead, Moncalvo, Vilgalys, Desjardin & B.A. Perry	HMJAU 43811	CHINA	MT497546	–	Unpublished
11	*A. aurantiidisca* (Murrill) Redhead, Moncalvo, Vilgalys, Desjardin & B.A. Perry	MF06837	USA	MT636967	–	Unpublished
12	*A. aurantiidisca* (Murrill) Redhead, Moncalvo, Vilgalys, Desjardin & B.A. Perry	DAOM216791	–	–	AF261360	Moncalvo et al. (2002)
13	*A. flavoalba* (Fr.) Redhead, Moncalvo, Vilgalys, Desjardin & B.A. Perry	604	ITALY	JF908464	–	Osmundson et al. (2013)
14	*A. flavoalba* (Fr.) Redhead, Moncalvo, Vilgalys, Desjardin & B.A. Perry	CBS 359.50	FRANCE	MH856659	MH868175	Vu et al. (2019)
15	*A. flavoalba* (Fr.) Redhead, Moncalvo, Vilgalys, Desjardin & B.A. Perry	CBS 258.53	FRANCE	MH857185	MH868723	Vu et al. (2019)
16	*A. flavoalba* (Fr.) Redhead, Moncalvo, Vilgalys, Desjardin & B.A. Perry	H6032608	FINLAND	MW540661	–	Unpublished
17	*A. flavoalba* (Fr.) Redhead, Moncalvo, Vilgalys, Desjardin & B.A. Perry	H6036822	FINLAND	MW540676	–	Unpublished
18	***A. flavida* Q. Na & Y.P. Ge**	**FFAAS03**50	**CHINA, Type**	**MW969653**	**MW969665**	**This study**
19	***A. flavida* Q. Na & Y.P. Ge**	**FFAAS0355**	**CHINA**	**MW969654**	–	**This study**
20	***A. rutila* Q. Na & Y.P. Ge**	**FFAAS0354**	**CHINA, Type**	**MW969658**	**MW969668**	**This study**
21	***A. rutila* Q. Na & Y.P. Ge**	**FFAAS0356**	**CHINA**	**MW969659**	–	**This study**
22	***A. taoyao* Q. Na & Y.P. Ge**	**FFAAS0351**	**CHINA**	**MW969655**	–	**This study**
23	***A. taoyao* Q. Na & Y.P. Ge**	**FFAAS0352**	**CHINA, Type**	**MW969656**	**MW969666**	**This study**
24	***A. taoyao* Q. Na & Y.P. Ge**	**FFAAS0353**	**CHINA**	**MW969657**	**MW969667**	**This study**
25	*Hemimycena albicolor* (A.H. Sm.) Elborne	MICH 11456	USA	MK169368	–	Unpublished
26	*H. gracilis* (Quél.) Singer	AFTOL–ID 1732	USA	DQ490623	DQ457671	Matheney et al. (2006)
27	*H. lactea* (Pers.) Singer	F33274	CANADA	MH718253	–	Unpublished
28	*H. lactea* (Pers.) Singer	MQ18R237–QFB30753	CANADA	MN992168	–	Unpublished
29	*H. mairei* (E.–J. Gilbert) Singer	CBS 263.47	FRANCE	MH856248	DQ457671	Vu et al. (2019)
30	*H. mairei* (E.–J. Gilbert) Singer	CBS 265.47	FRANCE	MH856249	MH867780	Vu et al. (2019)
31	*H. ochrogaleata* (J. Favre) M.M. Moser	409d	ITALY	JF908431	–	Osmundson et al. (2013)
32	*H. tortuosa* (P.D. Orton) Redhead	PDD:95759	NEW ZEALAND	HQ533011	–	Unpublished
33	*H. tortuosa* (P.D. Orton) Redhead	FRDBI 18076639	UK	MW487985	–	Unpublished
34	*Hydropus scabripes* (Murrill) Singer	GG355_86	NETHERLANDS	GU234149	–	Geml et al. (2009)
35	*Mycena abramsii* (Murrill) Murrill	231a	VENICE	JF908400	–	Osmundson et al. (2013)
36	*M. abramsii* (Murrill) Murrill	HMJAU 43282	CHINA	MH396626	–	Na and Bau (2019)
37	*M. abramsii* (Murrill) Murrill	HMJAU 43468	CHINA	MH396627	–	Na and Bau (2019)
38	*M. adscendens* Maas Geest.	Aronsen120803	NORWAY	KT900140	–	Larsson and Aronsen (2015)
39	*M. adscendens* Maas Geest.	Orstadius329–05	NORWAY	KT900141	–	Larsson and Aronsen (2015)
40	*M. adscendens* Maas Geest.	Aronsen061119	NORWAY	KT900142	–	Larsson and Aronsen (2015)
41	*M. adscendens* Maas Geest.	Aronsen120826	NORWAY	KT900143	–	Larsson and Aronsen (2015)
42	*M. alnetorum* J. Favre (=*M. abramsii* (Murrill) Murrill)	CM14–RG2	USA	KU295552	–	Unpublished
43	*M. amicta* (Fr.) Quél.	4745–HRL 1312	CANADA	KJ705188	–	Unpublished
44	*M. amicta* (Fr.) Quél.	CBS 352.50	FRANCE	MH856655	MH868170	Vu et al. (2019)
45	*M. amicta* (Fr.) Quél.	CBS 254.53	FRANCE	MH857183	–	Vu et al. (2019)
46	*M. amicta* (Fr.) Quél.	H6036851	FINLAND	MW540687	–	Unpublished
47	*M. arcangeliana* Bres.	252b	ITALY	JF908401	–	Osmundson et al. (2013)
48	*M. arcangeliana* Bres.	252f	ITALY	JF908402	–	Osmundson et al. (2013)
49	*M. cinerella* (P. Karst.) P. Karst.	Aronsen051014	SWEDEN	KT900146	–	Larsson and Aronsen (2015)
50	*M. cinerella* (P. Karst.) P. Karst.	173	RUSSIA	MF926553	–	Malysheva et al. (2017)
51	*M. citrinomarginata* Gillet	317h	ITALY	JF908416	–	Osmundson et al. (2013)
52	*M. citrinomarginata* Gillet	AD4TN	TUNISIA	KU973883	–	Unpublished
53	*M. clavicularis* (Fr.) Gillet	615i	ITALY	JF908466	–	Osmundson et al. (2013)
54	*M. clavicularis* (Fr.) Gillet	615b	ITALY	JF908467	–	Osmundson et al. (2013)
55	*M. diosma* Krieglst. & Schwöbel	KA13–1230	KOREA	KR673698	–	Kim et al. (2015)
56	*M. diosma* Krieglst. & Schwöbel	320f	ITALY	JF908417	–	Osmundson et al. (2013)
57	*M. entolomoides* T. Bau	HMJAU 43048	CHINA	MG654736	–	Na and Bau (2018)
58	*M. entolomoides* T. Bau	HMJAU 43052	CHINA	MG654737	–	Na and Bau (2018)
59	*M. entolomoides* T. Bau	HMJAU 43126	CHINA	MG654738	–	Na and Bau (2018)
60	*M. filopes* (Bull.) P. Kumm.	3782	FRANCE	KJ705175	–	Unpublished
61	*M. filopes* (Bull.) P. Kumm.	KA12–1699	KOREA	KR673631	–	Kim et al. (2015)
62	*M. filopes* (Bull.) P. Kumm.	287f	ITALY	JF908410	–	Osmundson et al. (2013)
63	*M. floridula* (Fr.) Quél. (=*Atheniella adonis*)	259	ITALY	JF908405	–	Osmundson et al. (2013)
64	*M. floridula* (Fr.) Quél. (=*Atheniella adonis*)	259a	ITALY	JF908406	–	Osmundson et al. (2013)
65	*M. floridula* (Fr.) Quél. (=*Atheniella adonis*)	CBS 360.50	FRANCE	MH856660	MH868176	Vu et al. (2019)
66	*M. floridula* (Fr.) Quél. (=*Atheniella adonis*)	HMJAU 43193	CHINA	MK309770	–	Unpublished
67	*M. floridula* (Fr.) Quél. (=*Atheniella adonis*)	HMJAU 43213	CHINA	MK309771	–	Unpublished
68	*M. floridula* (Fr.) Quél. (=*Atheniella adonis*)	HMJAU 43613	CHINA	MK309772	–	Unpublished
69	*M. galopus* (Pers.) P. Kumm.	BIOUG19840–F07	CANADA	MF908430	–	Dewaard (2017)
70	*M. leaiana* (Berk.) Sacc.	1028	ITALY	JF908376	–	Osmundson et al. (2013)
71	*M. leaiana* (Berk.) Sacc.	CNH03 (TENN)	USA	MF686520	–	Unpublished
72	*M. meliigena* (Berk. & Cooke) Sacc.	39	ITALY	JF908423	–	Osmundson et al. (2013)
73	*M. meliigena* (Berk. & Cooke) Sacc.	39d	ITALY	JF908429	–	Osmundson et al. (2013)
74	*M. metata* (Fr.) P. Kumm.	313b	ITALY	JF908412	–	Osmundson et al. (2013)
75	*M. olivaceomarginata* (Massee) Massee	GG436–86	NETHERLANDS	GU234119	–	Geml et al. (2012)
76	*M. olivaceomarginata* (Massee) Massee	CBS 228.47	FRANCE	MH856228	MH867756	Vu et al. (2019)
77	*M. olivaceomarginata* (Massee) Massee	CBS 229.47	FRANCE	MH856229	MH867757	Vu et al. (2019)
78	*M. olivaceomarginata* (Massee) Massee	HK47–15	NORWAY	MT153141	–	Thoen et al. (2020)
79	*M. pearsoniana* Dennis ex Singer	FCME25817	USA	JN182198	–	Harder et al. (2012)
80	*M. pearsoniana* Dennis ex Singer	TENN61544	USA	JN182199	–	Harder et al. (2012)
81	*M. pearsoniana* Dennis ex Singer	TENN61384	USA	JN182200	–	Harder et al. (2012)
82	*M. pelianthina* (Fr.) Quél.	CBH164	DENMARK	FN394548	–	Unpublished
83	*M. pelianthina* (Fr.) Quél.	108b	ITALY	JF908379	–	Osmundson et al. (2013)
84	*M. pelianthina* (Fr.) Quél.	108f	ITALY	JF908380	–	Osmundson et al. (2013)
85	*M. plumbea* P. Karst.	JN198391	CHINA	JN198391	–	Wu et al. (2013)
86	*M. plumbea* P. Karst.	420526MF0010	CHINA	MG719769	–	Wang et al. (2017)
87	*M. polygramma* (Bull.) Gray	439b	ITALY	JF908433	–	Osmundson et al. (2013)
88	*M. polygramma* (Bull.) Gray	439f	ITALY	JF908434	–	Osmundson et al. (2013)
89	*M. pura* (Pers.) P. Kumm.	TENN65043	USA	JN182202	–	Harder et al. (2012)
90	M. pura f. alba (Gillet) Kühner	CBH410	USA	FN394595	–	Unpublished
91	*M. purpureofusca* (Peck) Sacc.	F19748	CANADA	HQ604766	–	Unpublished
92	*M. purpureofusca* (Peck) Sacc.	G. Alfredsen	NORWAY	JQ358809	–	Unpublished
93	*M. rosea* Gramberg	938a	ITALY	JF908488	–	Osmundson et al. (2013)
94	*M. rosea* Gramberg	Champ–21	SPAIN	KX449424	–	Perez-Izquierdo et al. (2017)
95	*M. rubromarginata* (Fr.) P. Kumm.	407q	ITALY	JF908430	–	Osmundson et al. (2013)
96	*M. rubromarginata* (Fr.) P. Kumm.	TL–12780	DENMARK	KX513845	KX513849	Perry (2016)
97	*M. seminau* A.L.C. Chew & Desjardin	ACL136	MALAYSIA	KF537250	KJ206952	Chew et al. (2015)
98	*M. seminau* A.L.C. Chew & Desjardin	ACL308	MALAYSIA	KF537252	KJ206964	Chew et al. (2015)
99	*M. seynii* Quél.	71l	ITALY	JF908469	–	Osmundson et al. (2013)
100	*M. seynii* Quél.	71h	ITALY	JF908470	–	Osmundson et al. (2013)
101	*M. silvae–nigrae* Maas Geest. & Schwöbel	515	ITALY	JF908452	–	Osmundson et al. (2013)
102	*M. silvae–nigrae* Maas Geest. & Schwöbel	CC 13–12	USA	KF359604	–	Baird et al. (2014)
103	*M. stylobates* (Pers.) P. Kumm.	455	ITALY	JF908439	–	Osmundson et al. (2013)
104	*M. supina* (Fr.) P. Kumm.	128a	ITALY	JF908388	–	Osmundson et al. (2013)
105	*M. tenax* A.H. Sm.	p187i	USA	EU669224	–	Unpublished
106	*M. tenax* A.H. Sm.	OSC 113746	USA	EU846251	–	Unpublished
107	*M. vulgaris* (Pers.) P. Kumm.	447h	ITALY	JF908435	–	Osmundson et al. (2013)
108	*M. vulgaris* (Pers.) P. Kumm.	3781	CANADA	KJ705177	–	Unpublished
109	*M. zephirus* (Fr.) P. Kumm.	KA13–1265	KOREA	KR673722	–	Kim et al. (2015)

### Sequence alignment and phylogenetic analysis

A dataset comprising concatenated sequences for the ITS and nLSU regions from 45 accessions of three genera (*Atheniella*, *Hemimycena* Singer and *Mycena*) was compiled. A total of 112 sequences downloaded from GenBank and 11 sequences newly generated in this study were aligned and adjusted manually using BioEdit 7.0.4.1 and Clustal X (Thompson et al. 1997; Hall 1999). Gaps in the alignments were treated as missing data. The alignment was deposited with TreeBase (submission ID, 28111; study accession URL: http://purl.org/phylo/treebase/phylows/study/TB2:S28111). *Hydropus scabripes* (Murrill) Singer was chosen as the outgroup. The aligned dataset consisted of 836 ITS and 879 nLSU nucleotide sites (including gaps). The best-fit evolutionary model was determined using Modeltest 2.3 for each of the ITS and nLSU data partitions for Bayesian Inference (BI), which was implemented with MrBayes 3.2.6 (Ronquist and Huelsenbeck 2003; Nylander 2004). Markov Chain Monte Carlo (MCMC) chains sampling every 100^th^ generation until the topological convergence diagnostic value was less than 0.01 (Ronquist and Huelsenbeck 2003). Maximum Likelihood (ML) analysis was performed using raxmlGUI 1.5b1 and topological support was assessed using the rapid bootstrapping algorithm with 1000 replicates (Stamatakis et al. 2004). Topology support values, greater than 75% bootstrap support (ML) and 0.95 Bayesian posterior probabilities (BPP), are shown for relevant branch nodes.

## Results

### Phylogenetic analysis

**Figure 1.**
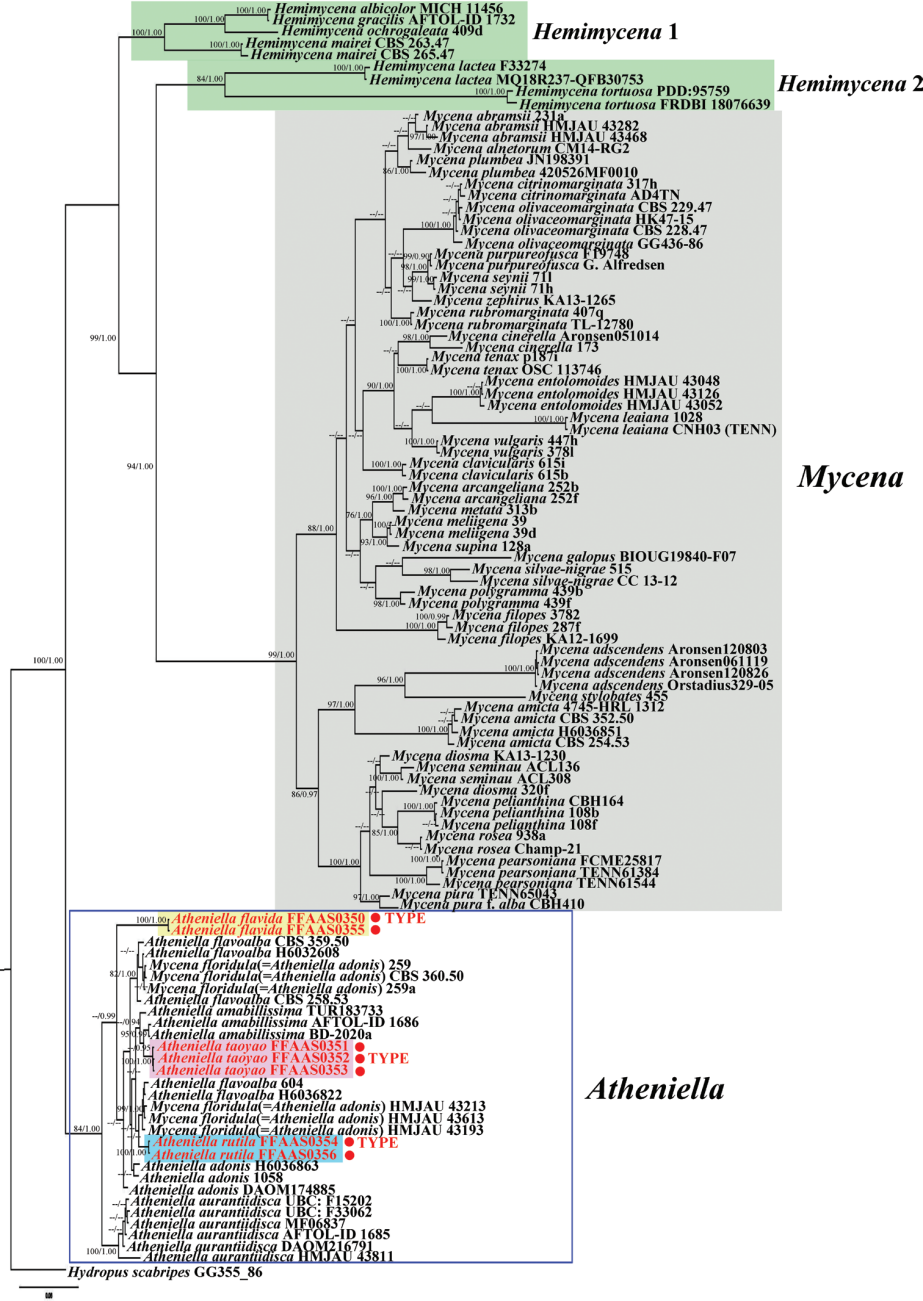
Maximum Likelihood and Bayesian tree concatenated ITS + nLSU dataset (ML ≥ 75%, BPP ≥ 0.90 are indicated). The tree is rooted with *Hydropus scabripes*. The new species are marked by red dot.

The concatenated dataset comprised 45 taxa and 1715 sites. The GTR + G evolutionary model was selected for both ITS and nLSU regions. The optimal evolutionary model for the 5.8S and nLSU partitions was lset nst = 6, rates = invgamma and prset statefreqpr = dirichlet (1,1,1,1). The BI and ML phylogenetic reconstructions were consistent in topology and, thus, only the BI tree is presented (Fig. 1).

The phylogenetic tree contained four major clades. Both *Atheniella* and *Mycena* were resolved as monophyletic. The six species of *Hemimycena* were resolved into two clades. Each of the four clades corresponded with high statistical support (ML bootstrap [BS] ≥ 84%, BI posterior probability [BPP] = 1).

The *Atheniella* Clade formed a sister group to the *Hemimycena* 1, *Hemimycena* 2 and *Mycena* clades with high statistical support (BS = 84%, BPP = 1.00). Samples of the three new species were placed in the *Atheniella* Clade and formed monophyletic lineages, each with high statistical support (*A. flavida*, BS = 100%, BPP = 1.00; *A. rutila*, BS = 100%, BPP = 1.00; *A. taoyao*, BS = 100%, BPP = 1.00; Fig. 1). The phylogenetic tree resolved *Atheniella flavida* as forming a monophyletic lineage, which was sister to the majority of accessions included within the *Atheniella* Clade, consisting of *A. adonis*, *A. amabillissima*, *A. flavoalba*, *Mycena floridula* and the other two new species. Recognition of *Atheniella taoyao* and *A. rutila* was well supported, with these two species respectively indicated to be sister to accessions of *A. amabillissima* and to accessions of *A. flavoalba* and *Mycena floridula* (Fr.) Quél. *Atheniella flavoalba* was polyphyletic with accessions placed in two distinct lineages together with accessions of *Mycena floridula*. Accessions of *Atheniella adonis* were distributed amongst three lineages and were difficult to distinguish genetically from accessions of *A. flavoalba* in two lineages.

### Taxonomy

#### Atheniella

Taxon classificationFungiAgaricalesMarasmiaceae

Redhead, Moncalvo, Vilgalys, Desjardin & B.A. Perry

96E7183A-B6A5-52F3-9A03-673A3A15CFD1

550101

##### Diagnosis.

Basidiomata small, mycenoid. Pileus conical, campanulate, to hemispherical, often with a small papilla when young, flattening or concave at centre with age; brightly coloured, white, creamy, yellow, orange, pinkish, reddish, sometimes yellow or deep brown at centre when old, the margin frequently fading to white, creamy, yellowish-white or yellow in the mature period; delicately pubescent, pruninose, glabrescent with age, translucent-striate, barely or shallowly sulcate, margin flattened and waved. Context thin and fragile, white. Lamellae ascending, adnate, adnexed, decurrent with tooth, faces concolorous with the sides. Stipe cylindrical, hollow, fragile, pruinose, almost smooth when old, base with coarse fibrils; white, yellow, orange, pink, sometimes base tinged deeper yellow with age. Odour and taste inconspicuous. Basidiospores globose, subglobose, ellipsoid, narrowly ellipsoid to cylindrical, smooth, thin-walled, hyaline, guttulate, inamyloid, white in prints. Basidia clavate, hyaline, thin-walled, 2- or 4-spored. Cheilocystidia fusiform, clavate, subutriform, long-stalked, hyaline, thin-walled. Pleurocystidia similar to cheilocystidia. Pileipellis hyphae covered with simple to branched excrescences, hyaline. Hyphae of the stipitipellis smooth or with simple cylindrical excrescences, hyaline; caulocystidia cylindrical, lageniform, subglobose, if present, hyaline, thin-walled. All tissues non-reactive in iodine. Clamps present or absent.

##### Habit and habitat.

Saprophytic on grass, moss, rotten wood or plant debris (leaves, pine needles and twigs).

##### Type species.

*Atheniella adonis* (Bull.) Redhead, Moncalvo, Vilgalys, Desjardin & B.A. Perry

##### Etymology.

Intentionally spelled to achieve phonetic harmony and uniqueness, the epithet alludes to the mythical goddess Athena (the combination of beautiful colouration, spear-like stature and shield-like pileus) and her ancient Mycenaean origin. Gender: feminine.

### Key to species of *Atheniella*

**Table:** 

1	Growing on twigs of *Filipendula ulmaria*	***A. ulmariae***
–	Growing on lawn or broadleaf-conifer mixed forest	**2**
2	Pileus yellowish-white, yellow to orange	**3**
–	Pileus pink or red	**7**
3	Cheilocystidia fusiform, thick-walled in the middle portion	***A. delectabilis***
–	Cheilocystidia fusiform, uniformly thin-walled	**4**
4	Clamps absent in all tissues	***A. flavida***
–	Clamps present in all tissues	**5**
5	Basidiospores broadly ellipsoid	***A. leptophylla***
–	Basidiospores narrowly ellipsoid	**6**
6	Caulocystidia up to 60 μm	***A. flavoalba***
–	Caulocystidia less than 20 μm	***A. aurantiidisca***
7	Lamellae decurrent	***A. taoyao***
–	Lamellae adnate to adnexed	**8**
8	Pileipellis with gelatinous hyphae	***Mycena rohitha* (≡ *A. rohitha*)**
–	Pileipellis without gelatinous hyphae	**9**
9	Cheilocystidia with several large irregular excrescences or otherwise nodulose	***Mycena wubabulna* (≡ *A. wubabulna*)**
–	Cheilocystidia entirely smooth	**10**
10	Stipe tinged coral-red and base yellowish with age	***A. amabillissima***
–	Stipe constantly white with age	**11**
11	Stipitipellis smooth; caulocystidia clavate to fusiform	***A. adonis***
–	Stipitipellis with simple cylindrical excrescences; caulocystidia not seen	***A. rutila***

#### Atheniella flavida

Taxon classificationFungiAgaricalesMarasmiaceae

Q. Na & Y.P. Ge sp. nov.

D60D42A0-3BEC-524A-B9F9-CB06F7CF1BD1

839378

Figs 2g–i , 3 , 4.

##### Diagnosis.

Pileus colour changing from orange-yellow to yellow, slightly concave at centre with age, pruninose. Lamellae narrowly adnate. Stipe densely pruinose. Basidiospores globose to subglobose, inamyloid. Cheilocystidia and pleurocystidia fusiform, thin-walled. Pileipellis with mass of excrescences. Caulocystidia cylindrical or lageniform. All tissues non-reactive in iodine. Clamps absent.

##### Holotype.

China. Yunnan Province, Yuxi City, Xinping County, Mopanshan National Forest Park, 25 Jul 2020, Qin Na, Yupeng Ge and Zewei Liu, *FFAAS0350* (Collection No. MY0182).

##### Etymology.

Refers to the yellow basidiomata.

##### Description.

Pileus 2.6–4.8 mm in diam., conic when young, becoming almost hemispherical and slightly concave at centre with age, orange-yellow (4A8) when young, fading to cream-yellow (3A4–3A6) at maturity, margin light yellow (3A3), sulcate, translucent-striate, delicately pubescent, pruninose, glabrescent with age, margin waved. Context very thin and fragile, pure white. Lamellae narrowly adnate, ascending, cream-white (3A2) to light yellow (3A3), faces concolorous with the sides, decurrent with a short tooth. Stipe slender, 5.5–12 × 0.5–0.8 mm, cylindrical, hollow, fragile, bright yellow (4A6), densely pruinose on the entire surface, almost smooth when old, base with sparse white fibrils. Odour and taste inconspicuous.

Basidiospores [60/3/2] (6.5) 6.7–**7.2**–7.8 (8.3) × (5.7) 5.9–**6.5**–7.1 (7.8) μm [Q = 1.03–1.22, **Q** = ***1.11*** ± 0.043] [holotype [40/2/1] (6.6) 6.7–**7.2**–7.6 (7.9) × (5.8) 5.9–**6.4**–6.9 (7.4) μm, Q = 1.04–1.20, **Q** = ***1.10*** ± 0.041], globose to subglobose, hyaline, guttulate, thin-walled, inamyloid. Basidia 20–29 × 5–8 μm, hyaline, clavate, 2-spored. Cheilocystidia abundant, 36–51 × 8–11 μm, fusiform, long-stalked, hyaline, thin-walled. Pleurocystidia similar to cheilocystidia, 28–43 × 6–10 μm. Pileipellis hyphae 2–6 μm wide, cutis; covered with mass of excrescences, 3.3–8.2 × 1.2–3.4 μm, hyaline. Hyphae of the stipitipellis 2–8 μm wide, hyaline, smooth; caulocystidia cylindrical or lageniform, 14–37 × 5–11 μm, hyaline, thin-walled. All tissues non-reactive in iodine. Clamps not seen in all tissues.

##### Habit and habitat.

Solitary to scattered on rotten wood in evergreen broad-leaf forest, *Cephalotaxus*, *Cunninghamia*, *Keteleeria*, *Podocarpus*, *Pseudotaxus*, *Pseudotsuga*, *Sequoia*, *Taxus*, *Torreya* and *Tsuga*.

##### Other specimens examined.

China. Yunnan Province, Yi Autonomous Prefecture, Chuxiong City, Zixishan, 27 Jul 2020, Qin Na, Yupeng Ge and Zewei Liu, *FFAAS0355* (Collection No. MY0234).

##### Remarks.

**Figure 2.**
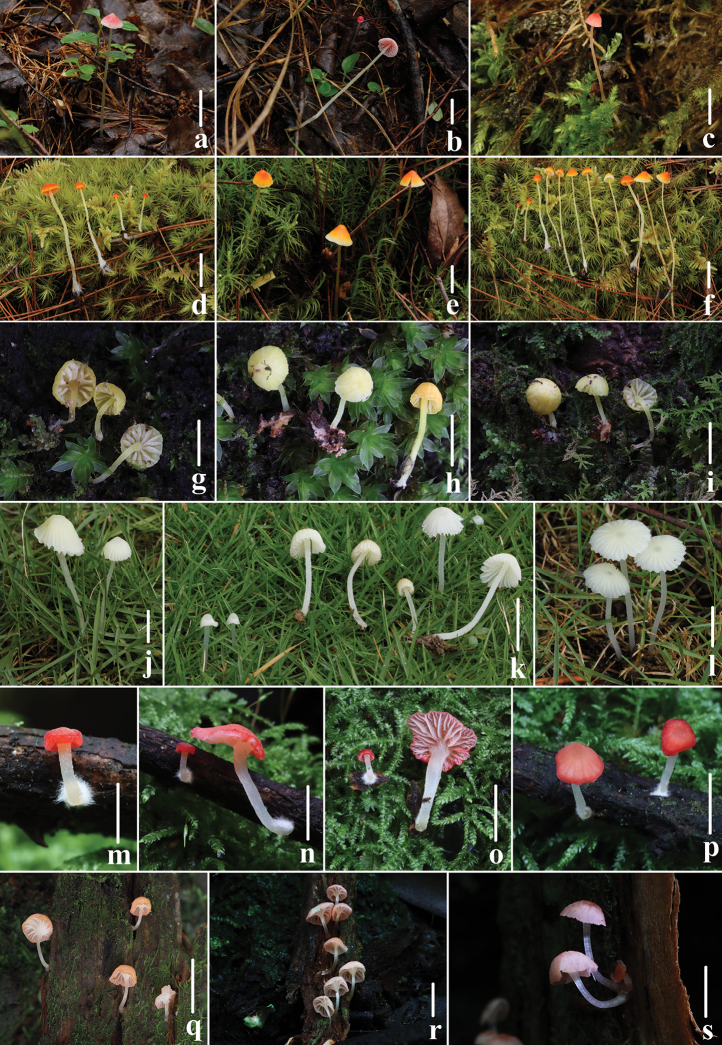
Basidiomata of *Atheniella* species **a–c***Atheniella adonis* (Bull.) Redhead, Moncalvo, Vilgalys, Desjardin & B.A. Perry **d–f***Atheniella aurantiidisca* (Murrill) Redhead, Moncalvo, Vilgalys, Desjardin & B.A. Perry **g–i***Atheniella flavida* Q. Na & Y.P. Ge **j–l***Atheniella flavoalba* (Fr.) Redhead, Moncalvo, Vilgalys, Desjardin & B.A. Perry **m–p***Atheniella rutila* Q. Na & Y.P. Ge **q–s***Atheniella taoyao* Q. Na & Y.P. Ge. Scale bars: 10 mm (**a–f, j–l, n–p**), 5 mm (**g–i, q–s**). Photographs **a,b, d–h, j–o, q, r** by Qin Na; **c, i, p, s** by Yupeng Ge.

*Atheniella flavida* is considered to be a distinct species in *Atheniella* on account of the pileus colour changing from orange-yellow to yellow, globose to subglobose basidiospores and caulocystidia comparatively small (Maas Geesteranus 1980, 1990, 1992a, 1992b; Perry 2002; Robich 2003; Aronsen and Læssøe 2016). Four species with a yellow or orange pileus are recorded: *A. aurantiidisca*, *A. delectabilis* (Peck) Lüderitz & H. Lehmann, *A. flavoalba* and *A. leptophylla* (Peck) Gminder & Böhningare (Smith 1935b; Maas Geesteranus 1980; Robich 2003; Aronsen and Læssøe 2016). *Atheniella flavoalba*, which is the most widely distributed species in the Northern Hemisphere, often seen in northeast China (Fig. 2a–c), shows the most morphological similarities to *A. flavida*; however, the former differs in forming cylindrical spores (6.5–9 × 3–4.5 μm) and the caulocystidia are fusiform and clavate to globose (Perry 2002; Robich 2003; Aronsen and Læssøe 2016; Na 2019). In contrast to *A. flavida*, *A. aurantiidisca*, which had been found in Yunnan Province and Tibet Autonomous Region of China (Fig. 2d–f) and *A. leptophylla* are easily mistaken for the new species (Robich 2003; Aronsen and Læssøe 2016; Na 2019). However, the pileus of *A. aurantiidisca* and *A. leptophylla* is constantly distinctly orange and caulocystidia of the two species are larger (up to 50 μm long) (Robich 2003; Aronsen and Læssøe 2016). *Atheniella delectabilis*, which was formerly named *Hemimycena delectabilis* (Peck) Singer on account of the white to yellowish-white pileus, decurrent lamellae and inamyloid basidiospores, is easily mistaken for *A. flavida* by the light yellowish pileus and the similar shape and size of cheilocystidia and caulocystidia. However, *A. delectabilis* is distinguishable from *A. flavida* by its decurrent lamellae and cylindrical spores (7–9 × 3–4 μm) (Smith 1935b; Malysheva and Morozova 2009). In addition, *A. delectabilis* produces cheilocystidia that are partially thick-walled (Smith 1935b).

**Figure 3.**
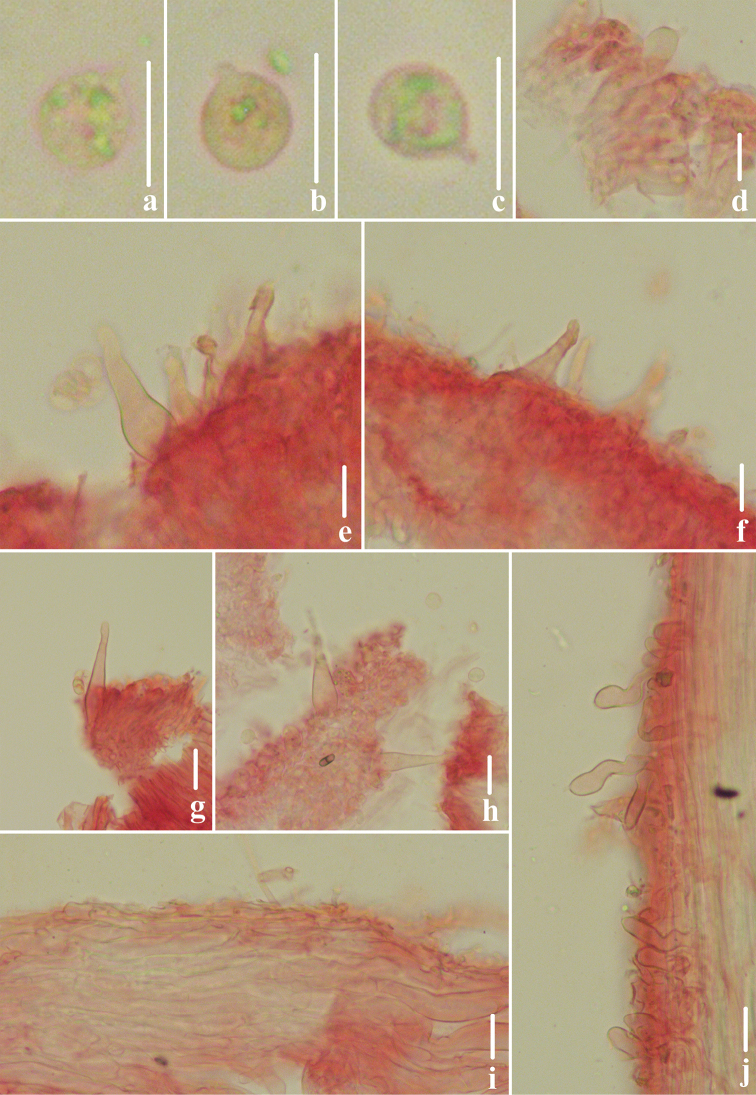
Microscopic features of *Atheniella flavida* (*FFAAS0350*, holotype) **a–c** basidiospores **d** basidia **e, f** cheilocystidia **g, h** pleurocystidia **i** pileipellis **j** stipitipellis and caulocystidia. Scale bars: 10 μm (**a–j**).

**Figure 4.**
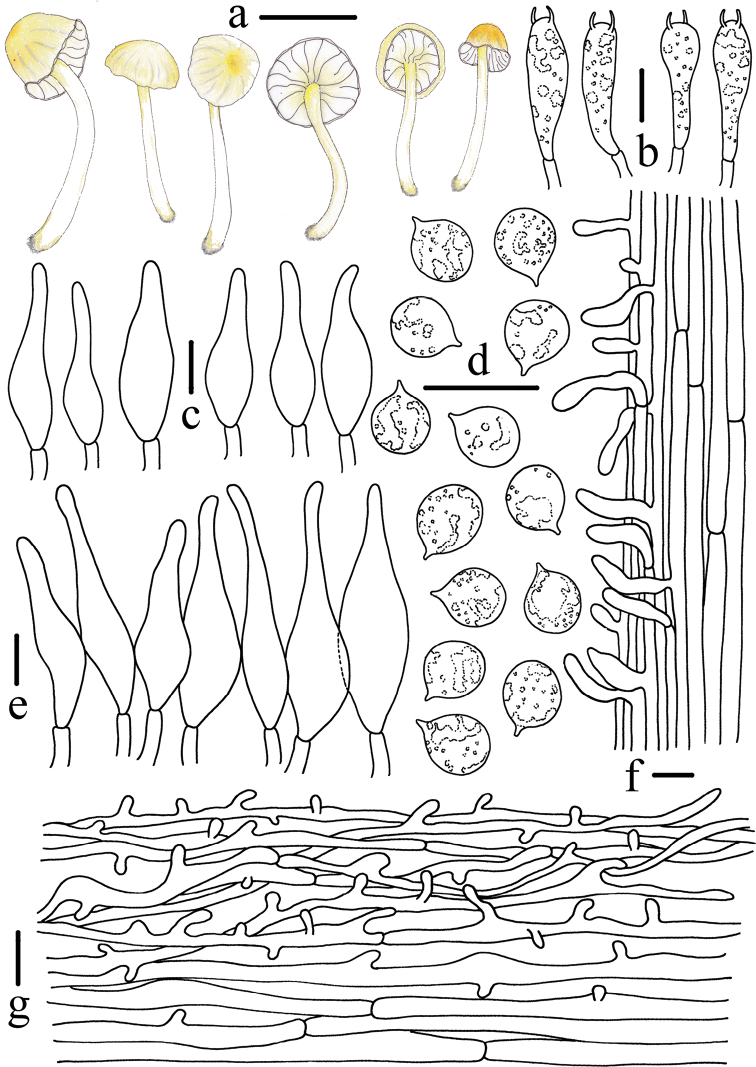
Morphological features of *Atheniella flavida* (*FFAAS0350*, holotype) **a** basidiomata **b** basidia **c** pleurocystidia **d** basidiospores **e** cheilocystidia **f** stipitipellis and caulocystidia **g** pileipellis. Scale bars: 10 mm (**a**); 10 μm (**b–g**). Drawings by Qin Na and Yupeng Ge.

#### Atheniella rutila

Taxon classificationFungiAgaricalesMarasmiaceae

Q. Na & Y.P. Ge sp. nov.

7B1AE07F-7AD5-5D1E-B577-2AEE315C0C95

839379

Figs 2m–p , 5 , 6.

##### Diagnosis.

Pileus campanulate to hemispherical, concave with age, slightly pruinose. Lamellae adnate to adnexed, white. Stipe base with dense white fibrils. Basidiospores cylindrical, inamyloid. Pleurocystidia similar to cheilocystidia, fusiform, with a long neck. Pileipellis covered with numerous excrescences. Hyphae of the stipitipellis with simple cylindrical excrescences. Caulocystidia not seen. All tissues non-reactive in iodine. Clamps absent.

**Figure 5.**
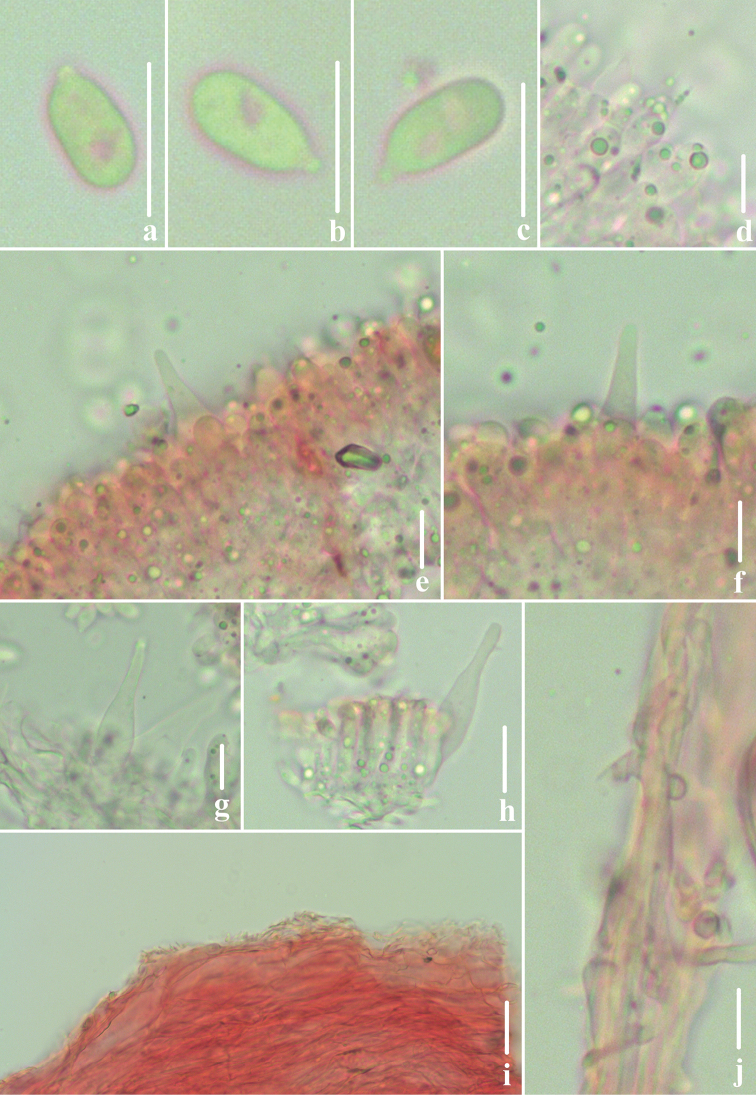
Microscopic features of *Atheniella rutila* (*FFAAS0354*, holotype) **a–c** basidiospores **d** basidia **e, f** cheilocystidia **g, h** pleurocystidia **i** pileipellis **j** stipitipellis. Scale bars: 10 μm (**a–j**).

**Figure 6.**
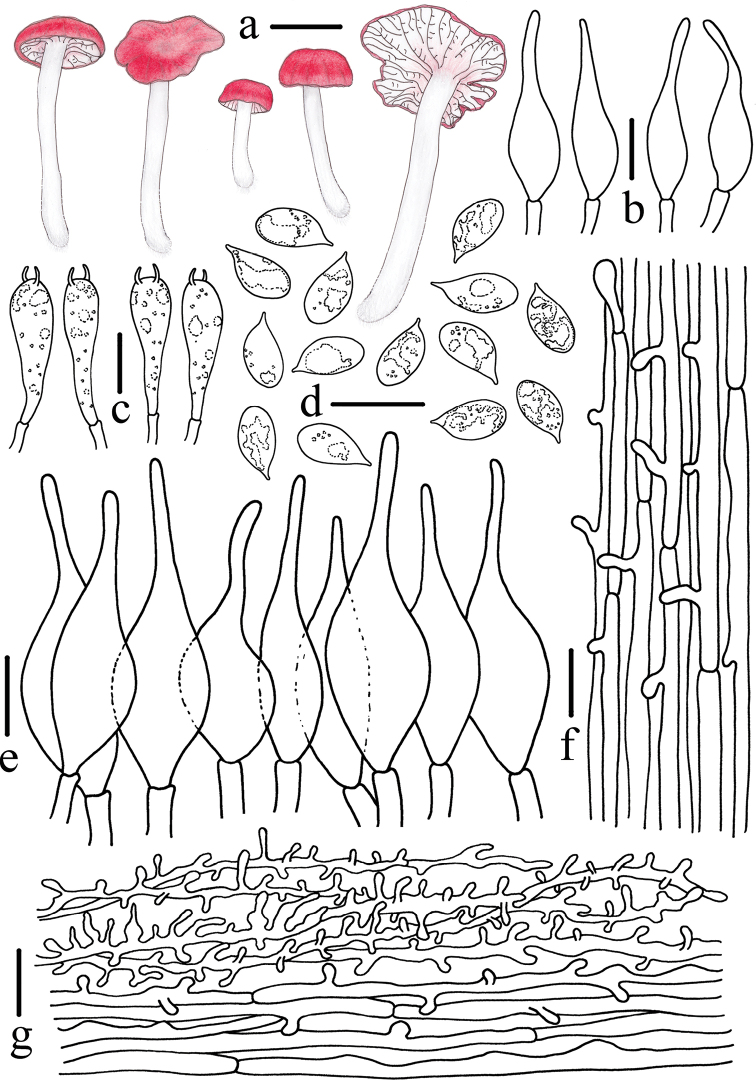
Morphological features of *Atheniella rutila* (*FFAAS0354*, holotype) **a** basidiomata **b** pleurocystidia **c** basidia **d** basidiospores **e** cheilocystidia **f** stipitipellis **g** pileipellis. Scale bars: 5 mm (**a**); 10 μm (**b–g**). Drawings by Qin Na and Yupeng Ge.

##### Holotype.

China. Yunnan Province, Lincang City, Wulaoshan National Forest Park, 31 Jul 2020, Qin Na, Yupeng Ge and Zewei Liu, *FFAAS0354* (Collection No. MY0210).

##### Etymology.

Refers to the bright red-tinted pileus.

##### Description.

Pileus 2.0–10.2 mm in diam., campanulate to hemispherical, applanate or slightly concave at centre when old, deep salmon (10A7) to bright red (10A8), shallowly sulcate, translucent-striate, delicately pubescent, glabrescent when old. Context white, thin, very fragile. Lamellae broadly adnate to adnexed, ascending, white, concolorous with the sides, basally interveined with age. Stipe 5.0–15.8 × 1.0–2.0 mm, cylindrical, hollow, fragile, transparent, pruninose, glabrescent when old, base slightly swollen, covered with dense white fibrils. Odour and taste indistinctive.

Basidiospores [60/3/2] (7.2) 7.7–**8.6**–9.8 (10.1) × (3.6) 4.1–**4.6**–5.3 (5.5) μm [Q = 1.71–2.05, **Q** = ***1.85*** ± 0.079] [holotype [40/2/1] (7.2) 7.5–**8.5**–9.7 (10.0) × (3.6) 4.1–**4.6**–5.2 (5.5) μm, Q = 1.72–1.99, **Q** = ***1.86*** ± 0.086], narrowly ellipsoid to cylindrical, hyaline in water and 5% KOH, inamyloid, smooth. Basidia 19–28 × 5–8 μm, 2-spored, clavate, hyaline. Cheilocystidia 32–45 × 8–11 μm, abundant, fusiform, with a long neck, thin-walled and hyaline. Pleurocystidia similar to cheilocystidia, 27–42 × 7–12 μm. Pileipellis hyphae 2–5 μm wide, covered with numerous excrescences, 3.2–6.9 × 0.8–1.7 μm, hyaline. Hyphae of the stipitipellis 2–7 μm wide, non-dextrinoid, hyaline, with simple cylindrical excrescences, 4.6–14.3 × 2.9–5.2 μm. All tissues non-reactive in iodine. Clamps absent in all tissues.

##### Habit and habitat.

Scattered on rotten wood in evergreen broadleaf and *Pinus* mixed forest.

##### Other specimens examined.

Yunnan Province, Puer City, Xiaoheijiang National Forest Park, 1 Aug 2020, Qin Na, Yupeng Ge and Zewei Liu, *FFAAS0356* (Collection No. MY0235).

##### Remarks.

*Atheniella rutila* is considered to be a distinct species in *Atheniella* on account of the bright red pileus, white stipe, narrowly ellipsoid to cylindrical and inamyloid spores and characters of the cystidia, pileipellis and stipitipellis (Maas Geesteranus 1980, 1990, 1992a, b; Perry 2002; Grgurinovic 2003; Robich 2003; Aravindakshan and Manimohan 2015; Aronsen and Læssøe 2016). *Atheniella amabillissima* is difficult to distinguish from *A. rutila* owing to the reddish basidiomata, but the pileus of *A. amabillissima* fades to white with age or has a dirty yellowish disc, and the spores are smaller (7–9 × 3–4 μm) (Smith 1935b). *Atheniella adonis* shows certain morphological similarities to *A. rutila* in possessing tiny and pinkish-red basidiomata, white lamellae and cylindrical basidiospores. However, *A. adonis* differs in producing a pileus with a white margin, longer stipe and clavate to fusiform caulocystidia (Perry 2002; Robich 2003; Aronsen and Læssøe 2016). In comparison with *Atheniella rutila*, *Mycena rohitha* (≡ *A. rohitha*) and *M. wubabulna* (≡ *A. wubabulna*) have gelatinous pileus hyphae and narrower basidiospores (Grgurinovic 2003; Aravindakshan and Manimohan 2015).

#### Atheniella taoyao

Taxon classificationFungiAgaricalesMarasmiaceae

Q. Na & Y.P. Ge sp. nov.

A96D1736-AF4F-5D87-9846-2408998FE214

839380

Figs 2q–s , 7 , 8

##### Diagnosis.

Pileus pinkish to light reddish. Lamellae decurrent. Stipe pruninose, base slightly swollen. Basidiospores narrowly ellipsoid to cylindrical, inamyloid. Cheilocystidia and pleurocystidia fusiform. Pileipellis hyphae covered with excrescences. Stipitipellis smooth, caulocystidia of two types, fusiform or subglobose. All tissues non-reactive in iodine. Clamps absent.

**Figure 7.**
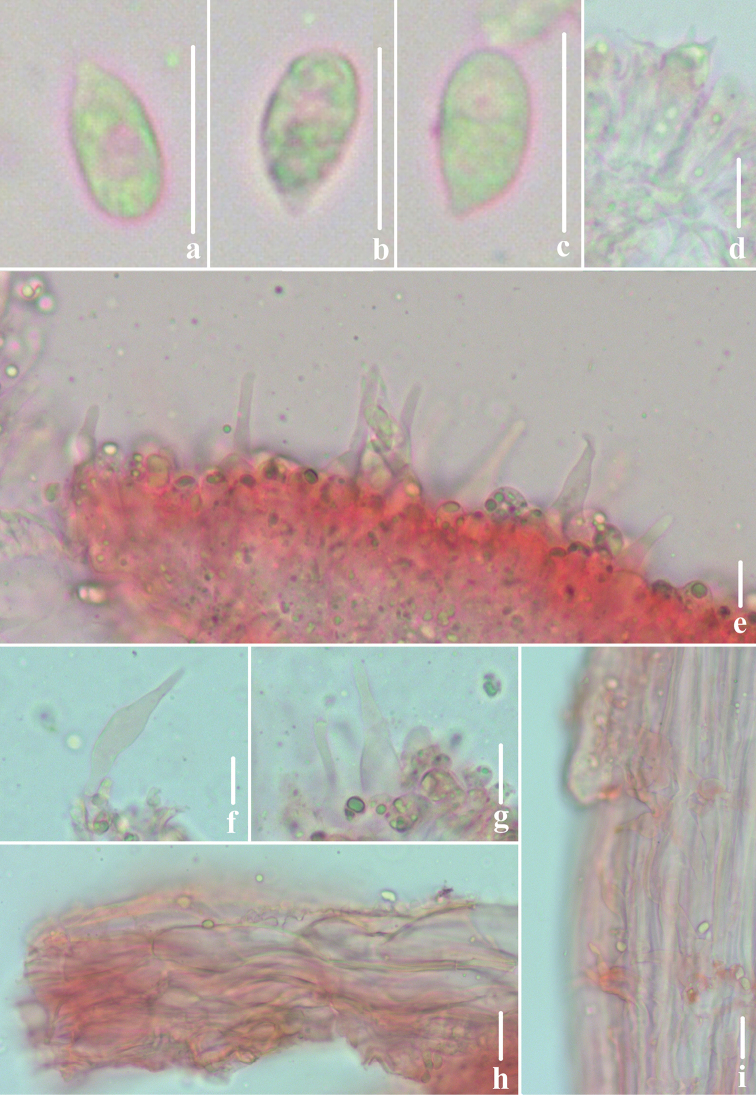
Microscopic features of *Atheniella taoyao* (*FFAAS0352*, holotype) **a–c** basidiospores **d** basidia **e** cheilocystidia **f, g** pleurocystidia **h** pileipellis **i** stipitipellis. Scale bars: 10 μm (**a– i**).

**Figure 8.**
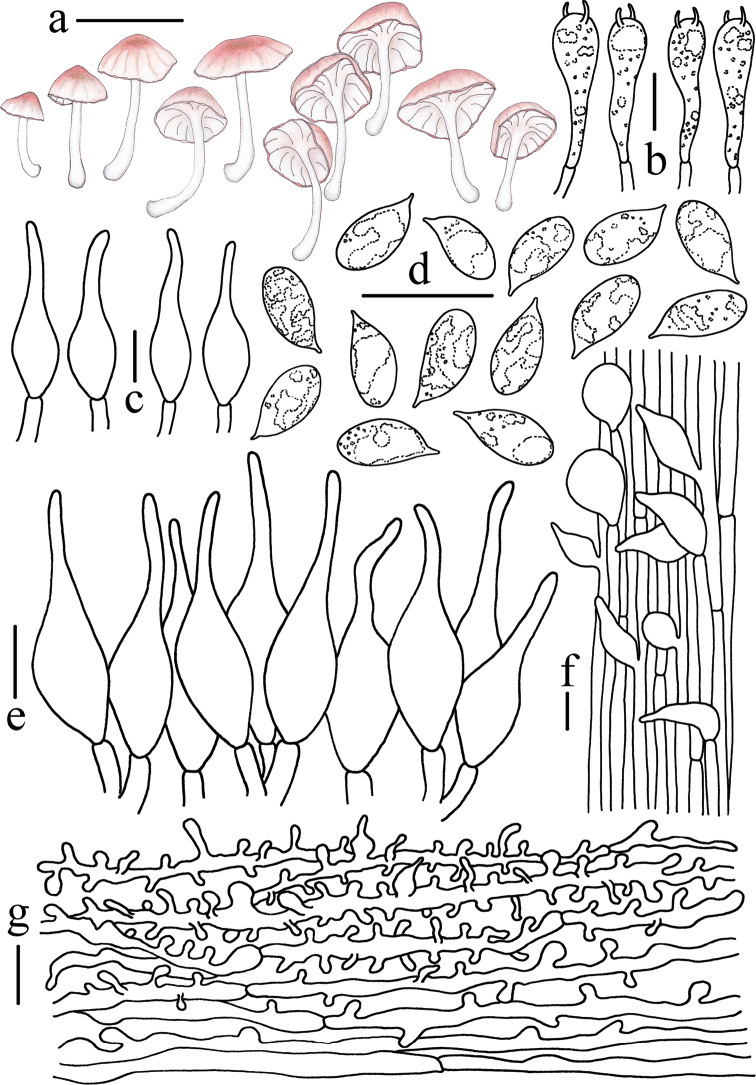
Morphological features of *Atheniella taoyao* (*FFAAS0352*, holotype) **a** basidiomata **b** basidia **c** pleurocystidia **d** basidiospores **e** cheilocystidia **f** stipitipellis and caulocystidia **g** pileipellis. Scale bars: 5 mm (**a**); 10 μm (**b–g**). Drawingsby Qin Na and Yupeng Ge.

##### Holotype.

China. Yunnan Province, Yuxi City, Xinping County, Mopanshan National Forest Park, 25 Jul 2020, Qin Na, Yupeng Ge and Zewei Liu, *FFAAS0352* (Collection No. MY0184).

##### Etymology.

Refers to the pinkish to reddish basidiomata. Tao Yao is a poem in the “The Book of Songs” that praises a young woman, whose beauty is compared to a flowering peach tree and who will be married and assume a new role in life.

##### Description.

Pileus 1.4–5.8 mm in diam., campanulate or hemispherical, obtusely umbonate in the centre, flattening with age, translucent-striate, light pink-salmon (8A3), light coral red (8B7), fading light pink (8A2) or white to the margin, delicately pubescent, glabrescent with age, with a flat margin. Context pure white, thin, fragile. Lamellae decurrent dentate, ascending, sparse, pure white, edges concolorous with the sides. Stipe 46–58 × 0.5–1.0 mm, central, terete, almost equal, hollow, fragile, transparent, pruninose, glabrescent with age, base slightly swollen, with tiny, white, fine hairs. Odourless, taste mild.

Basidiospores [80/4/3] (7.4) 7.7–**8.3**–9.1 (9.4) × (3.9) 4.1–**4.5**–5.0 (5.5) μm [Q = 1.73–2.08, **Q** = ***1.85*** ± 0.076] [holotype [40/2/1] (7.4) 7.7–**8.2**–9.0 (9.2) × (4.0) 4.1–**4.4**–5.0 (5.4) μm, Q = 1.75–1.99, **Q** = ***1.84*** ± 0.079], narrowly ellipsoid to cylindrical, hyaline, guttulate, thin-walled, inamyloid. Basidia 20–31 × 5–7 μm, hyaline, clavate, 2-spored. Cheilocystidia 23–42 × 5–10 μm, fusiform, long-stalked, hyaline. Pleurocystidia similar to cheilocystidia, 20–40 × 5–9 μm. Pileipellis hyphae 1–5 μm wide, cutis; covered with numbers of cylindrical or fusiform excrescences, 3.5–10.4 × 1.4–4.3 μm, hyaline. Hyphae of the stipitipellis 3–10 μm wide, hyaline, smooth; caulocystidia fusiform, 16.5–24.9 × 5.3–11.5 μm or subglobose, 11.8–16.5 × 9.1–12.9 μm. All tissues non-reactive in iodine. Clamps not seen.

##### Habit and habitat.

Scattered to gregarious on living wood in evergreen broadleaf forest, for example, *Cephalotaxus*, *Cunninghamia*.

##### Other specimens examined.

Yunnan Province, Yuxi City, Xinping County, Mopanshan National Forest Park, 25 Jul 2020, Qin Na, Yupeng Ge and Zewei Liu, *FFAAS0351* (Collection No. MY0183); Yunnan Province, Yuxi City, Xinping County, Shimenxia, 26 Jul 2020, Qin Na, Yupeng Ge and Zewei Liu, *FFAAS0353* (Collection No. MY0185).

##### Remarks.

*Atheniella taoyao* is unique in *Atheniella* because of the light pink-salmon pileus, decurrent lamellae and the two types of caulocystidia. *Atheniella adonis* most closely resembles *A. taoyao*, but the former differs in having adnate to adnexed lamellae, stipe with pink at the apex and larger caulocystidia (15–50 × 3.5–13.5 μm) (Aronsen and Læssøe 2016). *Atheniella amabillissima* is closely allied to *A. taoyao*, but differs in the larger basidiomata (pileus 3–20 mm in diam.), pileus fading to white or yellow with age, stipe tinted with coral red and yellow with age and the cheilocystidia are up to 65 μm in length (Smith 1935b). Aravindakshan and Manimohan (2015) described the species *Mycena rohitha* Aravind. & Manim. (≡ *Atheniella rohitha*) collected from India. This taxon differs from *Atheniella taoyao* in its orange stipe and gelatinous pileus hyphae (Aravindakshan and Manimohan 2015). *Mycena wubabulna*, a species described by Grgurinovic (2003) that should be transferred to *Atheniella*, is readily identified by its yellowish stipe base and cylindrical basidiospores (7.5–10.6 × 3.1–4.7 μm; Q = 2.3).

## Discussion

The present phylogenetic analysis showed that *Atheniella* formed a distinct clade independent of *Hemimycena* and *Mycena* with high BPP and BS support and, thus, supported segregation of the genus from the Mycenaceae (Moncalvo et al. 2002; Matheney et al. 2006). This finding also supported the view of Redhead et al. (2012) that *Atheniella*, formerly treated as Mycena sect. Adonideae, should be elevated to generic rank. *Atheniella* is more closely related to *Mycena* than to *Hemimycena*, based on genetic distance, in accordance with the greater similarity of *Atheniella* to *Mycena* spp. in morphological characters. The presence of pileocystidia and the morphological differences of the cheilocystidia, caulocystidia and stipitipellis can be used to distinguish *Atheniella* species from *Hemimycena* and *Mycena*.

*Atheniella* was originally established by Redhead et al. (2012) to accommodate four species: *A. adonis*, *A. amabillissima*, *A. aurantiidisca* and *A. flavoalba*. In recent years, the number of recognised species of *Atheniella* has increased to nine, but the description of the genus was incomplete and not detailed (Redhead et al. 2012; Gminder and Böhning 2016; Lehmann and Lüderitz 2019). With description of the new species in the present study, the generic description for *Atheniella* requires updating. Amongst *Atheniella* species, the bright colour of the pileus may be uniform or be tinted at the centre, but fades to white at the margin, the lamellae are adnate to decurrent and the stipe colour sometimes changes to yellow or pink towards the base. With regards to micromorphological characters, *Atheniella* produces globose to cylindrical spores, caulocystidia are present or absent and the stipitipellis is smooth or has projections.

*Atheniella* is closely allied to *Hemimycena*, Mycena sect. Aciculae Kühner ex Singer and Mycena sect. Oregonenses Maas Geest., based on morphology (Maas Geesteranus 1980, 1992a, b). Species of *Hemimycena* lack bright yellow, pink to red basidiomata, produce larger spores and pileocystidia are often seen (Antonín and Noordeloos 2004; Malysheva and Morozova 2009). *M. acicula* (Schaeff.) P. Kumm., which is the sole species classified in Mycena sect. Aciculae, shares an orange-coloured pileus, non-amyloid spores and ornamentation of the pileipellis, but the stipitipellis is covered with numerous excrescences and is embedded in gelatinous material (Maas Geesteranus 1990; Robich 2003; Aronsen and Læssøe 2016). A longer stipe (up to 60 mm) with yellow fibrils at the base, cheilocystidia fusiform or lageniform with a rounded apex and caulocystidia with yellow contents are morphological characters that distinguish Mycena sect. Oregonenses from *Atheniella* (Maas Geesteranus 1990).

Morphological and molecular evidence support classification of the three newly-recognised species as members of *Atheniella*. The three species share white lamellae, a pruninose stipe base without a basal disc, inamyloid basidiopores, fusiform and thin-walled cheilocystidia, pileipellis covered with excrescences and are unreactive in Melzer’s Reagent. In addition, the three species grow on rotten wood or other plant debris. *A. flavida* is mainly distinguished from *A. taoyao* and *A. rutila* by its distinctly yellowish-orange to yellow pileus and globose spores. The pinkish or reddish basidiomata support the inclusion of *A. taoyao* and *A. rutila* in *Atheniella*. Compared with *A. rutila*, *A. taoyao* is readily discriminated, based on the light pink basidiomata, narrow ellipsoid basidiospores and subglobose or fusiform caulocystidia. *A. amabillissima* shows the most morphological similarities to *A. taoyao* and *A. rutila*; however, *A. amabillissima* has a pileus which fades to white with age, smaller spores and longer cheilocystidia (Smith 1935b).

It is noteworthy that the taxonomic status of *Mycena floridula* remains unresolved (Josserand 1930; Kühner 1938; Aronsen and Læssøe 2016). This species was formerly classified in Mycena sect. Adonideae as a form of *M. flavoalba* with a pink pileus (Josserand 1930; Kühner 1938; Aronsen and Læssøe 2016). More recently, it was proposed that the name *M. floridula* was a synonym of *A. adonis* (Redhead et al. 2012). The phylogenetic reconstructions in our study and accessions of *M. floridula* indicated that *M. floridula* was closely related to *A. flavoalba*, with little genetic distinction between the two taxa. Therefore, we tentatively accept *M. floridula* as a pink form of *A. flavoalba*, but emphasise that a detailed appraisal of the morphological and molecular variation of *A. flavoalba* is required.

## Supplementary Material

XML Treatment for Atheniella

XML Treatment for Atheniella flavida

XML Treatment for Atheniella rutila

XML Treatment for Atheniella taoyao
